# Relationship between sleep disturbance and developmental status in preschool-aged children with developmental disorder

**DOI:** 10.1186/s12887-024-04857-1

**Published:** 2024-05-30

**Authors:** Sung Hyun Kim, Chung Reen Kim, Donghwi Park, Kang Hee Cho, Je Shik Nam

**Affiliations:** 1 Department of Rehabilitation Medicine, Bundang Jesaeng General Hospital, Seongnam, South Korea; 2 Department of Physical Medicine and Rehabilitation, Ulsan University Hospital, University of Ulsan College of Medicine, Ulsan, South Korea; 3 Department of Rehabilitation Medicine, Daegu Fatima Hospital, Daegu, South Korea; 4 Department of Rehabilitation Medicine, Chungnam National University Hospital, Chungnam National University College of Medicine, 282 Munhwa-ro, Jung-gu Daejeon, Daejeon, 35015 South Korea

**Keywords:** Sleep, Sleep-Wake Disorders, Preschool Child, Child Development, Developmental Disabilities

## Abstract

**Background:**

Sleep has been known to affect childhood development. Sleep disturbance is likely more common in children with developmental delay (DD) than in typical development. There are few studies on the correlation between sleep disturbance and developmental features in children with DD. Therefore, this study aimed to evaluate the associations between the two in children with DD.

**Methods:**

A total of 45 children (age range 27.0 ± 11.1) with DD were recruited and evaluated using the Sleep Disturbance Scale for Children (SDSC) and Bayley Scales of Infant and Toddler Development (BSID-III). The outcomes are expressed as means and standard deviations. The correlation between SDSC and BSID-III was assessed using Spearman’s rank correlation test. Multiple regression analysis was performed to investigate the relationship between BSID-III domains and SDSC questionnaire subscales. Statistical significance was set at *p* < 0.05.

**Results:**

Based on the correlation analysis and subsequent hierarchical regression analysis, cognition and socio-emotional domains of BSID-III were significantly associated with the DOES subscale of the SDSC questionnaire. In addition, the expressive language domain of the BSID-III was found to be associated with the DA subscale of the SDSC questionnaire. It seems that excessive daytime sleepiness might negatively affect emotional and behavioral problems and cognitive function. Also, arousal disorders seem to be related to memory consolidation process, which is thought to affect language expression.

**Conclusion:**

This study demonstrated that DA and DOES subscales of the SDSC questionnaire were correlated with developmental aspects in preschool-aged children with DD. Sleep problems in children with DD can negatively affect their development, thereby interfering with the effectiveness of rehabilitation. Identifying and properly managing the modifiable factors of sleep problems is also crucial as a part of comprehensive rehabilitation treatment. Therefore, we should pay more attention to sleep problems, even in preschool-aged children with DD.

## Introduction

Sleep disorders are common in infants and children. Approximately 20–30% of children worldwide, including Korea suffer from sleep disorders [1–3]. The prevalence of sleep disorders is more common in children with developmental delay (DD) than those with typical development, and its nature is known to be long-term and more complex [4, 5]. 

According to the International Classification of Sleep Disorders-3 (ICSD-3), seven categories are identified as a sleep disorder: insomnia disorders, sleep-related breathing disorders, central disorders of hypersomnolence, circadian rhythm sleep–wake disorders, sleep-related movement disorders, parasomnias, and other sleep disorders [6]. 

Among these, difficulties initiating or maintaining sleep and sleep-disordered breathing are the common types of sleep orders in children with DD [4]. 

Sleep disorders can have harmful effects on physical health, mood, cognitive impairment, and academic performance [4, 7]. 

Sleep has been known to affect childhood development and learning because it facilitates infant generalization and enhances precise memory after 18–24 months of age [8]. 

Several studies have verified the association between sleep and motor development in infants and toddlers. S. Manacero and M. L. Nunes conducted a study on healthy preterm infants with low birth weight, showing that delayed development was correlated with sleep quality [9]. X Liang et al. showed the bidirectional relationship between sleep problems and infant development in infants younger than six months old using longitudinal data [10]. In addition, Z Zhang et al. demonstrated sleep consolidation is correlated with cognitive impairment, and longer, persistent sleep duration could aid gross motor development in toddlers [11]. 

While these studies were conducted on children who are typically developing or with DD over time, to the best of the authors’ knowledge, studies regarding the correlation between sleep disturbance and developmental aspects in children with DD receiving rehabilitation treatment are still lacking. Thus, only children diagnosed with DD were enrolled, and sleep disturbance was hypothesized to be correlated with development in children with DD receiving one or more rehabilitation treatments.

## Methods

### Study design

This cross-sectional study was conducted in a single rehabilitation center between December 2021 and June 2022. The study protocol was approved by the Institutional Review Board and the Ethics Committee of Bundang Jesaeng General Hospital in accordance with the Declaration of Helsinki (no. 2023-07-005). The written informed consent form was obtained from each child’s parents or guardians for the study participation, as all of the children in this study were younger than 18 years of age.

### Participants

Children diagnosed with DD who had participated in a rehabilitation program through admission or outpatient treatment were recruited. DD refers to slower-than-expected progress in the attainment of developmental milestones that are typically achieved by a certain age.

Participants were recruited through paper-based advertisements in the Department of Rehabilitation Medicine of a general hospital and through direct contact with potential participants who have previously expressed interest in participating in clinical research.

Among them, participants were included if they met the following inclusion criteria: (1) children ages between 18 and 42 months, (2) children who underwent developmental evaluation within six months before and after assessing sleep disturbance assessment using the SDSC questionnaire, and (3) children whose legal guardian agreed to participate in this study. Children who were previously diagnosed with sleep-related symptoms or had a history of receiving medication were excluded.

### Outcome variables

#### Assessment of sleep quality in children

The Sleep Disturbance Scale for Children (SDSC) questionnaire was used as a tool to assess sleep disturbances in toddlers. It was originally developed for school-aged children between the ages of 6 and 15 years and has proven to have good psychometric validity [12]. Recently, the validity of the SDSC questionnaire translated into other country languages was demonstrated in preschool-aged children [13]. 

It consisted of 26 items divided into six subscales: disorders of initiating and maintaining sleep (DIMS), sleep breathing disorders (SDB), disorders of arousal (DA), sleep-wake transition disorders, disorders of excessive somnolence (DOES), and sleep hyperhidrosis (SHY).

These items were rated by their parents on a 5-point Likert scale for children’s sleep patterns over the past six months.

To accurately convey the meaning of the English questionnaire to caregivers, we decided to translate it into Korean. One of the authors first translated the English version of the SDSC questionnaire into Korean. It was translated only in easy-to-understand Korean, and no further changes were made. Then, the Korean version of the SDSC was checked for ease of understanding by the caregivers and other physiatrists of our study.

#### Assessment of the developmental status

##### Bayley scales of infant and toddler development *(BSID-III)*

BSID is one of the extensive development assessment tools for diagnosing DDs in young children aged between 1 and 42 months [14]. It comprises five domains: cognition, language (receptive and expressive), motor (gross motor and fine motor), socio-emotional scale, and adaptive behavior.

We used the Korean version of BSID-III, which was found to be suitable for application to Korean infants and toddlers after verification studies of reliability and validity in a preliminary study. When performing the test, cognitive, language, and motor domains are evaluated by the physical therapist. On the other hand, the social-emotional domain is designed so that the primary caregiver or parent answers questions on the questionnaire [15, 16]. 

### Statistical analysis

All data were analyzed using SPSS statistics version 26.0 (IBM, Chicago, IL, USA). The significance level was set to *p* < 0.05. The Shapiro–Wilk test was used to determine the normality of the data. Descriptive statistics with continuous normal distribution are presented as mean ± standard deviation (SD).

Correlation coefficients between the SDSC questionnaires and other variables were calculated using Spearman’s rank correlation coefficient.

Multiple variables regression analysis was performed to control potential confounding factors and determine the independent contribution of subscales of SDSC questionnaires to outcome variables.

## Results

### Demographic characteristics of participants

This study included 45 children (27 boys and 18 girls). Their mean age was 27 months (SD = 11.1; range 10–48 months). Among them, 11 participants were children diagnosed with cerebral palsy (CP), while the remaining participants were diagnosed with other developmental disorders (Table 1).

**Table 1:** Baseline characteristics of the participants

	Values
**Total number**	45
**Gender (male/female)**	27:18
**Age (months)**	27.0 ± 11.1
**CP: non-CP NDI**	11:34
SDSC*	
DIMS	61.7 ± 13.4
SBD	54.9 ± 8.6
DA	48.5 ± 4.6
SWT	61.1 ± 11.2
DOES	51.3 ± 12.0
SHY	59.3 ± 14.9
Total	61.6 ± 13.2
**BSID-III†**	
Cognition	6.1 ± 4.2
Receptive language	6.2 ± 3.6
Expressive language	5.2 ± 3.0
Gross motor	4.0 ± 3.7
Fine motor	5.7 ± 3.7
Socio-emotional	6.6 ± 3.9

Values are presented as means ± standard deviations

CP: cerebral palsy; non-CP NDI: non-cerebral palsy neurodevelopmental impairment; DIMS, difficulty in initiating and maintaining sleep; SBD: sleep breathing disorder; DA: disorders of arousal; SWT: sleep-wake transition disorder; DOES: disorders of excessive somnolence; SHY: sleep hyperhidrosis; BSID-III: Bayley scales of infant and toddler development III

*scores based on the T score

†scores based on the scale ranging from 1 to 19

Children with non-cerebral palsy neurodevelopmental impairment include premature infants without brain lesions, genetic diseases including CHARGE syndrome, Prader willi syndrome, Down syndrome, and Shone’s syndrome. Also, children with general developmental delay but who were not included in the criteria for cerebral palsy were also included.

### Relationship between the SDSC T scores and BSID-III

We found a statistically significant relationship between the disorders of arousal (DA) subscale of the SDSC questionnaire and the expressive language domains of the BSID-III with a Spearman coefficient of *r* = 0.309 (*p* < 0.05).

Our study also revealed that the DOES subscale of the SDSC questionnaire was significantly related to cognition, receptive language, fine motor, and socio-emotional domains of the BSID-III. Spearman’s coefficient of r for each domain was as follows: − 0.343 (*p* < 0.05) for cognition, − 0.33 (*p* < 0.05) for receptive language, − 0.324 (*p* < 0.05) for fine motor, and –0.0456 (*p* < 0.01) for socio-emotional domains of the BSID-III.

In addition, total scores of the SDSC questionnaire were significantly related to socio-emotional domains with a Spearman coefficient of *r* = − 0.436 (*p* < 0.01) (Table 2).

**Table 2:** Spearman’s correlation analysis of Age, sex, SDSC questionnaire subscales, and BSID-III domains

Variables	Age	Sex	DIMS_ T score	SBD_ T score	DA_ T score	SWTD_ T score	DOES_ T score	SHY_ T score	Total_ T score	Cognition	Receptive language	Expressive language	FM	GM	SE
**Age**	1														
**Sex**	0.054	1													
**DIMS_** **T score**	0.295	−0.193	1												
**SBD_** **T score**	−0.19	0.001	0.318^*^	1											
**DA_** **T score**	0.091	−0.159	0.363^*^	0.154	1										
**SWTD_** **T score**	0.085	−0.16	0.587^***^	0.39^**^	0.474^**^	1									
**DOES_** **T score**	−0.013	0.341^*^	0.326^*^	0.324^*^	0.126	0.343^*^	1								
**SHY_** **T score**	0.063	−0.078	0.463^**^	0.065	0.049	0.393^**^	0.28	1							
**total_** **T score**	0.125	−0.058	0.831^***^	0.504^***^	0.367^*^	0.774^***^	0.658^***^	0.61^***^	1						
**Cognition**	0.048	−0.258	−0.213	−0.171	0.166	−0.098	−0.343*	−0.18	−0.271	1					
**Receptive** **language**	0.198	−0.148	−0.199	−0.132	0.102	−0.124	−0.33*	−0.083	−0.243	0.864^***^	1				
**Expressive** **language**	0.019	−0.068	−0.152	−0.054	0.309*	−0.021	−0.25	−0.089	−0.159	0.831^***^	0.854^***^	1			
**Fine motor**	−0.062	−0.31^*^	−0.162	−0.119	0.164	−0.097	−0.324*	−0.105	−0.213	0.914^***^	0.796^***^	0.802^***^	1		
**Gross** **Motor**	−0.09	−0.237	0.088	0.064	0.015	0.139	−0.138	0.089	0.081	0.602^***^	0.493^***^	0.529^***^	0.665^***^	1	
**SE**	−0.068	−0.197	−0.322*	−0.239	0.026	−0.28	−**0.0456** **	−0.247	−0.436 **	0.721***	0.606***	0.58***	0.683***	0.44 ***	1

* *p* < 0.05 ** *p* < 0.01 *** *p* < 0.001

* DIMS: difficulty in initiating and maintaining sleep; SBD: sleep breathing disorder; DA: disorders of arousal; SWT: sleep-wake transition disorder; DOES: disorders of excessive somnolence; SHY: sleep hyperhidrosis; FM: fine motor; GM: gross motor, SE : socio-emotional

### Association between the SDSC questionnaire subscales and BSID-III domains

Based on the results of correlation analysis using Pearson correlation coefficients,

hierarchical regression analysis was conducted in order of high correlation, and the resulting model is depicted in Table 3.

**Table 3:** Association of subscales of SDSC questionnaire with domains of BSID-III by multiple regression analysis

Variables	Cognition			Receptive Language			Expressive Language			Fine motor			Gross motor			Socio-emotional		
	B	ß	*p*	B	ß	*p*	B	ß	*p*	B	ß	*p*	B	ß	*p*	B	ß	*p*
**DIMS_T score**	−0.067	–0.215	0.180	−0.047	–0.175	0.285	−0.051	–0.230	0.145	−0.043	–0.157	0.334	0.043	0.155	0.372	−0.072	–0.255	0.098
**DA_T score**	0.261	0.283	0.066	0.160	0.204	0.194	**0.274**	**0.421**	0.007**	0.210	0.259	0.098	−0.015	–0.018	0.913	0.141	0.169	0.246
**DOES_T score**	−**0.108**	–**0.309**	0.043*	−0.089	–0.298	0.056	−0.056	–0.228	0.126	−0.094	–0.305	0.049*	−0.058	–0.187	0.255	−0.125	–0.395	0.008**
**(Constant)**	3.130		0.633	5.904		0.308	−2.019		0.658	2.971		0.614	4.989		0.434	10.652		0.066
***R*** ^***2***^	0.199			0.154			0.222			0.167			0.039			0.267		
***Adjusted R*** ^***2***^	0.140			0.092			0.222			0.106			−0.031			0.213		

* *p* < 0.05 ** *p* < 0.01 *** *p* < 0.001

B : Unstandardized coefficient, **β** : Standardized coefficient

In multiple regression analysis, the DOES subscale of the SDSC questionnaire showed a significant negative correlation with cognition (β = − 0.309, *p* < 0.05) and socio-emotional domains of the BSID-III (β = –0.395, *p* < 0.01).

The DA subscale of the SDSC questionnaire was found to have a significant positive correlation with expressive language domains of the BSID-III (β = 0.421, *p* < 0.01).

On the other hand, the receptive language domain of BSID-III did not show statistically significant correlations with the DOES (β = -0.298, *p* = 0.56) and DA (β = 0.204, *p* = 0.194) subscale of the SDSC questionnaire. Similarly, the fine motor domain of BSID-III did not exhibit statistically significant correlations with the DOES (β = -0.305, *p* = 0.049, F = 2.733, *p* > 0.05), indicating non-significance. Additionally, there were no significant correlations observed between the gross motor domain of BSID-III and the DOES (β = -0.187, *p* = 0.255) and DA (β = -0.018, *p* = 0.913) subscale of the SDSC questionnaire (Table 3).

## Discussion

In this study, in the SDSC questionnaires, DA and DOES subscales were significantly related to developmental aspects (cognition, fine motor, language, and social awareness) in children with DD.

However, tests for gross-motor function and functional abilities were not associated with SDSC questionnaires.

Several studies have evaluated the relationships between sleep problems and developmental aspects in typically developing infants and toddlers [9, 10]. 

Studies regarding sleep problems in children with developmental problems have also been reported [5, 17]. Additionally, some studies have assessed the relationship between sleep problems and their associated developmental aspects [18–20]. However, these studies are generally limited to specific disease groups, such as autism spectrum disorder and cerebral palsy.

Our study verified sleep disorders in children with developmental delay and found a relationship with developmental delay, but on the contrary, there is research showing that sleep disorder affects brain development. Leong et al. suggested that sleep plays an important role in cognition; sleep and sleep-related cognitive processing may be influenced by brain development [21]. 

To the best of our knowledge, researches regarding these relationships in toddlers and preschoolers with DD, including various disease entities, have yet to be published. For preschool-aged children, their school life is the beginning of socialization and is vital for development. Therefore, we aimed to evaluate the relationships between the two targeting children during this period. Moreover, we conducted the study using assessment tools commonly used in clinical practice for an extended period not designed for research so that parents’ understanding of the tool could be warranted, which is crucial in identifying children’s developmental status.

The study demonstrated a significant correlation between DA in the SDSC questionnaire and language expression in BSID-III, with DA appearing to impact language expression in children with DD negatively.

Generally, sleep is considered to have an important role in language learning and communication skills [22]. It may be required for long-term retention after learning and improvement in the acquisition and integration of vocabulary in school-aged children [23]. 

The day-and-night sleep duration ratio was also correlated with language outcomes, suggesting that early mature sleep consolidation could be helpful in preventing language delays in developing children [24]. 

In addition, sleep cycles or stages are thought to influence language learning, such as vocabulary acquisition [25]. For example, in previous research regarding the relationship between sleep disturbance and language deficits in toddlers with DS, the authors suggested that a specific consolidation process during slow-wave sleep (SWS) might contribute to these correlations based on their findings that difficulties with expressive language such as vocabulary and syntax were observed in children with DS with poor sleep quality [23]. 

According to the International Classification of Sleep Disorders, third edition (ICSD-3), sleepwalking and night terrors are subtypes of NREM parasomnia and correspond to the disorder of arousal.

The DA domain of the SDSC questionnaire contains not only NREM sleep parasomnias but also nightmares that mainly occur during REM sleep. A study demonstrated that individuals experiencing nightmares exhibit poor sleep quality and altered sleep architectures, including relatively shortened SWS and NREM duration [26]. 

Although the exact mechanism is not clearly understood, it might be related to the language learning process and recall, thereby affecting the language expression. Further research will be needed to establish a more precise understanding of this relationship.

Our study also demonstrated a notable correlation between DOES of the SDSD questionnaire and the socio-emotional and cognition domains of BSID-III, respectively.

This result suggests that excessive somnolence might be an important factor that negatively affects the development of children.

Excessive somnolence, a type of hypersomnia, is a condition characterized by falling asleep during the day repeatedly and excessively. It can be caused by sleep deprivation, obstructive sleep apnea, sedating medications, and medical, psychiatric, or neurologic disorders [27]. 

Previous studies demonstrated that sleep problems in children associated with excessive daytime sleepiness (EDS) could cause various adverse effects, which include emotional and behavioral problems, cognitive function impairments, academic performance, and health-related concerns such as injuries, drug abuse, endocrine function, which are in the similar context as the results of our study [28, 29]. 

A recent systemic review demonstrated that although few studies are included, the association between sleep problems and cognition and behavior was identified, and this could be observed early as a preschool-aged child [30]. 

Several studies have been reported regarding the relationship between EDS and social behavior in preschool children.

A recent cross-sectional study in Italy found that EDS was negatively correlated with prosocial behaviors and could affect teacher–student relationships in kindergarten children [31]. In addition, a national population study conducted with Australian preschool-aged children indicated a connection between sleep problems and health-related quality of life (HRQoL), particularly in the psychosocial aspect. Notably, morning tiredness was associated with the poorest HRQoL [32]. 

However, Goodlin-Jones B et al. demonstrated somewhat different results, which indicates that in contrast to nighttime sleep problems, daytime sleepiness didn’t show a significant relationship with a decline in daytime performance in preschool-aged children [33]. 

As objectively measuring DES is difficult, especially in preschool-aged children, and mostly relying on questionnaires or parental reports, this might lead to potential measurement error and could be the reason for the somewhat different study results.

However, based on past studies on the behavioral consequences of sleep problems,

It is thought that sleep problems can cause excessive stress, aggression, and problems with attention and concentration, which can lead to problems with social behavior, such as academic performance or adaptation to school life [7, 34]. 

Meanwhile, DD itself seems to precipitate sleep disorders. More children with DD experience sleep disturbances than typically developing children [4]. 

The rates and nature of sleep difficulties are different among the groups of children with DDs. However, regardless of the groups, their sleeping difficulties might be related to their behavioral difficulties [35]. 

Our study revealed that DA subscales of the SDSC questionnaires have a positive relationship with the expressive language of BSID-III. While sleep problems associated with arousal may appear to have a negative impact on language development, we hypothesize that children with better expressive language skills may communicate their sleep-related arousal issues more effectively to their caregivers. This aspect may have been reflected in the questionnaire responses. Studies with a large number of participants will be warranted to establish a precise causal relationship between these.

We believe that our study could have the following significance. From the perspective of caregivers, previous articles have shown that children’s age, weekly hours of rehabilitation therapy, and burden level of caregivers show a negative and positive correlation, respectively [36]. 

As the subjects of this study were preschool children receiving rehabilitation treatment, the burden level of their parents might be high. Also, many caregivers of children with DD could be exposed to sleep disturbance due to caregiving [37, 38]. 

By paying attention to sleep problems in children with DD and providing appropriate management, the burden levels and health problems, including the sleep quality of caregivers, can be alleviated.

This study has potential limitations.

First, as it is a cross-sectional study, the temporal relationship between sleep disorders and children’s development cannot be determined, and thus, the long-term effects of variables cannot be assessed.

Second, factors such as medication were not controlled, so the possibility that such factors may have affected sleep and development, respectively, cannot be ruled out.

Third, the study was limited by a relatively small sample size and limited heterogeneity of DD causes. To generalize the relationship between development and sleep disturbance, further studies with a larger number of patients with DD, including each cause of DD, are necessary.

Fourth, in preschool-aged children, objective sleep measurement is difficult; therefore, most studies primarily rely on reports from or questionnaire answers of their caregivers or teachers. Thus, future research using objective methods to measure the quality and quantity of sleep in children will be needed.

Lastly, The Korean version of the SDSC questionnaire that we used has not been proven for validity and reliability by previous studies. Though we tried to convey the meaning of the English questionnaire in Korean as much as possible, differences in meaning could occur.

A validation study of the Korean version of the questionnaire will be necessary in future research.

## Conclusion

This study demonstrated a correlation between sleep disturbance and development in infants and toddlers with DD. Among the sleep domains, DA and DOES are correlated with developmental aspects in preschool-aged children diagnosed with DD, with the former being significantly associated with the expression domains of the BSID-III while the latter demonstrating a correlation with cognitive and socio-emotional scales.

Considering the effects of sleep on a child’s development, timely intervention to minimize sleep disturbance is necessary and considered to be helpful for the development of children with DD.

## Data Availability

The data are available from the corresponding author on reasonable request.

## Abbreviations

BSID-III: Bayley scales of infant and toddler development
SDSC: Sleep disturbance scale for children
DOES: Disorders of excessive somnolence
DA: Disorders of arousal
